# Glycoprotein Acetyls: A Novel Inflammatory Biomarker of Early Cardiovascular Risk in the Young

**DOI:** 10.1161/JAHA.121.024380

**Published:** 2022-02-12

**Authors:** Scott T. Chiesa, Marietta Charakida, Georgios Georgiopoulos, Justin D. Roberts, Simon J. Stafford, Chloe Park, Juha Mykkänen, Mika Kähönen, Terho Lehtimäki, Mika Ala‐Korpela, Olli Raitakari, Milla Pietiäinen, Pirkko Pussinen, Vivek Muthurangu, Alun D. Hughes, Naveed Sattar, Nicholas J. Timpson, John E. Deanfield

**Affiliations:** ^1^ Institute of Cardiovascular Science University College London UK; ^2^ Department of Imaging Science and Biomedical Engineering King’s College London UK; ^3^ Cambridge Centre for Sport and Exercise Sciences Anglia Ruskin University Cambridge UK; ^4^ Molecular Diagnostics Unit Medical Technology Research Centre Faculty of Health, Education, Medicine & Social Care Anglia Ruskin University Chelmsford UK; ^5^ Cardiometabolic Phenotyping Group Institute of Cardiovascular Science University College London UK; ^6^ Research Centre of Applied and Preventive Cardiovascular Medicine University of Turku Finland; ^7^ Centre for Population Health Research University of Turku and Turku University Hospital Finland; ^8^ Department of Clinical Physiology Tampere University Hospital Tampere Finland; ^9^ Finnish Cardiovascular Research Center Tampere Faculty of Medicine and Health Technology Tampere University Tampere Finland; ^10^ Department of Clinical Chemistry Fimlab Laboratories Tampere Finland; ^11^ Computational Medicine Faculty of Medicine University of Oulu and Biocenter Oulu Finland; ^12^ Center for Life Course Health Research University of Oulu Finland; ^13^ NMR Metabolomics Laboratory School of Pharmacy University of Eastern Finland Kuopio Finland; ^14^ Department of Clinical Physiology and Nuclear Medicine Turku University Hospital Turku Finland; ^15^ Oral and Maxillofacial Diseases University of Helsinki and Helsinki University Hospital Helsinki Finland; ^16^ Centre for Cardiovascular Imaging UCL Institute of Cardiovascular Science London United Kingdom; ^17^ MRC Unit for Lifelong Health and Ageing University College London UK; ^18^ Institute of Cardiovascular and Medical Sciences British Heart Foundation (BHF) Glasgow Cardiovascular Research Centre University of Glasgow UK; ^19^ Population Health Sciences Bristol Medical School Faculty of Health Sciences University of Bristol UK; ^20^ Medical Research Council Integrative Epidemiology Unit University of Bristol UK

**Keywords:** ALSPAC, cardiovascular disease, CRP, GlycA, Young Finns Study, Cardiovascular Disease, Risk Factors

## Abstract

**Background:**

Low‐grade inflammation in the young may contribute to the early development of cardiovascular disease. We assessed whether circulating levels of glycoprotein acetyls (GlycA) were better able to predict the development of adverse cardiovascular disease risk profiles compared with the more commonly used biomarker high‐sensitivity CRP (C‐reactive protein).

**Methods and Results:**

A total of 3306 adolescents and young adults from the Avon Longitudinal Study of Parents and Children (mean age, 15.4±0.3; n=1750) and Cardiovascular Risk in Young Finns Study (mean age, 32.1±5.0; n=1556) were included. Baseline associations between inflammatory biomarkers, body composition, cardiovascular risk factors, and subclinical measures of vascular dysfunction were assessed cross‐sectionally in both cohorts. Prospective risk of developing hypertension and metabolic syndrome during 9‐to‐10‐year follow‐up were also assessed as surrogate markers for future cardiovascular risk. GlycA showed greater within‐subject correlation over 9‐to‐10‐year follow‐up in both cohorts compared with CRP, particularly in the younger adolescent group (r=0.36 versus 0.07). In multivariable analyses, GlycA was found to associate with multiple lifestyle‐related cardiovascular disease risk factors, cardiometabolic risk factor burden, and vascular dysfunction (eg, mean difference in flow‐mediated dilation=−1.2 [−1.8, −0.7]% per z‐score increase). In contrast, CRP levels appeared predominantly driven by body mass index and showed little relationship to any measured cardiovascular risk factors or phenotypes. In both cohorts, only GlycA predicted future risk of both hypertension (risk ratio [RR], ≈1.1 per z‐score increase for both cohorts) and metabolic syndrome (RR, ≈1.2–1.3 per z‐score increase for both cohorts) in 9‐to‐10‐year follow‐up.

**Conclusions:**

Low‐grade inflammation captured by the novel biomarker GlycA is associated with adverse cardiovascular risk profiles from as early as adolescence and predicts future risk of hypertension and metabolic syndrome in up to 10‐year follow‐up. GlycA is a stable inflammatory biomarker which may capture distinct sources of inflammation in the young and may provide a more sensitive measure than CRP for detecting early cardiovascular risk.

**Nonstandard Abbreviations and Acronyms**
ALSPAC: Avon Longitudinal Study of Parents and Children
GlycA: glycoprotein acetyls
YFS: Young Finns Study

**Clinical Perspective**

**What Is New?**

- Low‐grade inflammation may contribute towards the early progression of atherosclerotic cardiovascular disease during young adulthood, decades before the onset of clinical symptoms; however, previous studies attempting to link inflammation to early cardiovascular risk in the young using the well‐established inflammatory biomarker high‐sensitivity CRP (C‐reactive protein) have produced mixed results.
- We assessed whether circulating levels of a novel nuclear magnetic resonance‐derived biomarker of systemic inflammation—glycoprotein acetyls—were better able to predict the early development of adverse cardiovascular disease risk profiles when compared with CRP.

**What Are the Clinical Implications?**

- Low‐grade inflammation captured by the novel biomarker glycoprotein acetyls is associated with adverse cardiovascular risk profiles from as early as adolescence and predicts future risk of hypertension and metabolic syndrome in up to 10‐year follow‐up.
- Glycoprotein acetyls is a stable inflammatory biomarker which may capture distinct sources of inflammation in the young and may provide a more sensitive measure than CRP for detecting and stratifying early cardiovascular risk.

Chronic low‐grade inflammation is a hallmark of both cardiometabolic and cardiovascular disease and is most commonly quantified in clinical research using high‐sensitivity assays of CRP (C‐reactive protein). Although likely not a causal risk factor for disease itself,^1^ CRP lies downstream of multiple inflammatory pathways implicated in numerous chronic conditions, and has repeatedly been shown to predict risk of type 2 diabetes and cardiovascular disease (CVD) in later‐life.^2^, ^3^

While clinical events attributed to atherosclerotic CVD predominantly occur from mid‐life onwards, their appearance represents the culmination of a decades‐long disease process which may start virtually from childhood.^4^ Whether chronic low‐grade inflammation contributes to the emergence of the early subclinical signs of disease in the time before mid‐life remains unclear, however, largely because of a lack of randomized clinical trials at this age. In cross‐sectional analyses, some—but not all—studies have reported modest associations between CRP and various surrogate markers of early disease risk such as hypertension, metabolic syndrome, and vascular dysfunction.^5^, ^6^, ^7^, ^8^, ^9^, ^10^, ^11^ However, these associations have not been replicated in Mendelian Randomization studies,^12^, ^13^ and are often attenuated to null in studies where appropriate adjustments are made for potential confounders at this young age such as obesity.^5^, ^7^, ^9^ These findings may suggest one of 2 things—(1) that inflammation predominantly contributes to the CVD process in the later stages of disease, and early associations may simply result from confounding by obesity, or (2) that different inflammatory pathways to those upstream of CRP may underlie adverse changes at an earlier stage of disease evolution, and that novel biomarkers may therefore be required for monitoring low‐grade inflammatory burden at this age.

Recent research has identified a potential role for a nuclear magnetic resonance (NMR)‐derived measure of glycoprotein acetylation—termed GlycA—as a novel biomarker of systemic inflammation.^14^ The GlycA NMR signal represents the integrated concentration and glycosylation of numerous acute phase proteins (predominantly alpha‐1‐acid glycoprotein, haptoglobin, and alpha‐1‐antitrypsin) released in the inflammatory state. This novel biomarker has been shown in multiple cohorts of older individuals to predict the future development of both type 2 diabetes and CVD independently of CRP,^15^, ^16^ suggesting that it may capture different upstream inflammatory pathways relevant to both diseases. No study to‐date, however, has investigated the relationship between GlycA and early subclinical manifestations of disease, or assessed its effectiveness for predicting the risk of future adverse outcomes in the young.

Using 2 large and extensively phenotyped longitudinal cohorts comprising both adolescents and young adults, we now report the first findings relating GlycA and CRP to the emergence of cardiovascular risk profiles in early‐life and investigate the ability of each to predict vascular and adverse cardiovascular risk profiles in up to 10‐year follow‐up.

## Methods

### Data Availability

Both the ALSPAC (Avon Longitudinal Study of Parents and Children) and YFS (Young Finns Study) cohorts operate a system of managed open access for qualified researchers wishing to access study data. Further details on each can be found under ‘Detailed Cohort Descriptions’ in Data S1.

### Study Population

Participants were drawn from 2 ongoing longitudinal cohorts of young people based in the UK and Finland (ALSPAC and YFS, respectively). ALSPAC is a prospective birth cohort study investigating factors that influence normal childhood development and growth, whereas YFS is a multi‐center follow‐up study conducted in 5 Finnish cities (Helsinki, Kuopio, Oulu, Tampere, and Turku) and their rural surroundings. Cohort and study designs for both studies have been described in detail previously,^17^, ^18^, ^19^, ^20^ and a brief description of each is provided in Data S1. In ALSPAC, ethical approval was obtained from the ALSPAC Ethics and Law Committee and Local Research Ethics Committees. If the child was younger than age 16 years at the time of consent, informed written consent was obtained from the parent/guardian alongside assent from the child. When age ≥16 years, all participants provided their own informed written consent. In YFS, written informed consent was obtained from local research ethics committees. All ethical approvals from both cohorts conformed to the Declaration of Helsinki and all biological samples used in the study were collected in accordance with the Human Tissue Act (2004).

### Study Design

The ability of each inflammatory biomarker to predict cardiovascular risk in the transition from adolescence to young adulthood was tested in ALSPAC using data collected from individuals who attended both Teen Focus 3 (mean age, 15 years) and Focus at 24 (mean age, 24 years) clinics. Study data were collected and managed using REDCap electronic data capture tools hosted at the University of Bristol.^21^ REDCap (Research Electronic Data Capture) is a secure, web‐based software platform designed to support data capture for research studies. Additional measures of vascular function and fat distribution were also collected in a subset of these individuals (n=379) attending a specialist vascular clinic at age 21 years. Data collected from YFS participants attending both 2001 (mean age, 32 years) and 2011 (mean age, 42 years) follow‐up clinics were subsequently used to test the same hypotheses during the transition from early‐ to mid‐adulthood, with additional measures of vascular function also assessed at a separate 2007 clinic (mean age, 37 years). Only participants with both inflammatory markers measured at baseline and with data on hypertension and metabolic syndrome status at follow‐up were included. Unless otherwise stated, any participants with CRP levels >10 mg/L were excluded from analyses to reduce the risk of confounding arising from acute infection, leaving a total of 3306 individuals (ALSPAC n=1750 and YFS n=1556) in the study.

### Inflammatory, Cardiometabolic, and Lifestyle‐Related Risk Factors

GlycA in both studies was measured as part of an NMR metabolomics platform (Nightingale Health, Helsinki, Finland) as described elsewhere.^22^ High‐sensitivity CRP was measured by automated particle‐enhanced immunoturbidimetric assay in ALSPAC (Roche UK, Welwyn Garden City, UK), and by an Olympus AU400 with “CRP‐UL” assay kit in YFS (Wako Chemicals, Neuss, Germany). Body mass index (BMI) was calculated as weight(kg)/height(m)^2^ and waist circumference was measured using a flexible tape to the nearest 1 mm at the midpoint between the lower ribs and the iliac crest. In ALSPAC, fat and lean mass were measured by dual energy X‐ray absorptiometry in participants aged 15 years using a Lunar Prodigy narrow fan‐beam densitometer. In a subset of individuals at age 21 years, visceral and subcutaneous adipose volumes were further quantified using magnetic resonance imaging as previously described.^23^ Lipopolysaccharide and the ratio of LBP/sCD14 (lipopolysaccharide‐binding protein to soluble cluster of differentiation 14) were measured as markers of gut‐derived inflammation using ELISA assays (Hycult Biotech, Immunodiagnostik Oy, Finland). Blood lipids, glucose, and insulin were all measured as previously described,^24^ and insulin resistance was estimated using the Homeostasis Model Assessment 2 for Insulin Resistance (HOMA2‐IR, Diabetes Trials Unit, Oxford). Smoking and alcohol intake were assessed by self‐recall questionnaires administered to participants at their baseline visit, with smoking then dichotomized as never/ever smoked, and alcohol intake as ≤2 drinks/week versus >2 drinks/week. In ALSPAC, highest household occupation was used to assign participants a household social class using the 1991 British Office of Population Census Statistics classification,^25^ and physical activity was objectively measured using average counts per minute recorded over 7 days via an MTI Actigraph AM7164 2.2 accelerometer. In YFS, socioeconomic position was assessed using current occupational status (manual/lower‐grade non‐manual/higher‐grade non‐manual), and physical activity using a physical activity index generated from questionnaire data detailing exercise habits and frequency, as previously described.^26^

### Vascular Outcomes

Systolic and diastolic blood pressures were measured in the seated position in ALSPAC and supine position in YFS using automated sphygmomanometers at ages 15, 21, and 24 years in ALSPAC and 32, 37, and 42 years in YFS. Mean arterial pressure was calculated as diastolic blood pressure+((systolic blood pressure‐diastolic blood pressure/3)). Additional vascular phenotyping of carotid intima‐media thickness and flow‐mediated dilation were also performed in both the subset of ALSPAC participants attending the 21‐year clinic and in all YFS participants at the 37‐year clinic. Further details of these vascular phenotyping techniques are available in Data S1.

### Longitudinal Cardiovascular Risk

In the absence of hard CVD end points at this young age, 2 complementary and well‐established surrogate markers of subclinical disease measured at ages 24 years (ALSPAC) and 42 years (YFS) were used for longitudinal analyses—namely hypertension and metabolic syndrome. Full details of how each of these were classified can be found in Data S1.

### Statistical Analysis

Continuous data were summarized as mean±SD or median (interquartile range) if skewed. Normal distribution was assessed using the Shapiro–Wilk test alongside graphical inspection of histograms and normality plots, and non‐normally distributed data were natural log‐transformed before inclusion in statistical models.

#### Associations With Lifestyle‐Related Factors

To assess independent associations of fat and lean mass to biomarker levels, each independent variable was stratified into tertiles before being grouped into 9 groups (high lean/low fat through to high fat/low lean). ANCOVA models were used to test for effects across groups after adjustment for a wide range of potentially confounding factors: namely age, sex, BMI, waist circumference, triglycerides, high‐density lipoprotein cholesterol, glucose, diastolic blood pressure, physical activity, and socioeconomic status. Associations with different fat distributions were next tested using multivariable linear regression models in a subset of patients with magnetic resonance imaging phenotyping at age 21 years. For this analysis, both subcutaneous and visceral fat mass (as exposures) and inflammatory biomarkers (as outcomes) were converted to z‐scores to allow direct comparisons between tests. All models were adjusted for the opposing fat mass (ie, subcutaneous for visceral and vice‐versa) alongside the covariates mentioned previously. To test associations with gut‐derived inflammatory markers, multivariable regression analyses with similar adjustments were again used to estimate the z‐score increase in each biomarker per z‐score increase in LBP/sCD14. Next, ANCOVA models were used to test for associations between physical activity and inflammatory biomarker levels, both in isolation and in conjunction with levels of BMI. For the former, objective measures of physical activity were split into quartiles for the exposure, while for the latter, a similar approach to that used when testing fat/lean mass was used (ie, 9 groups ranging from low fat/high fit to high fat/low fit). Similar models were also used to test for associations with smoking status (never/ever), alcohol intake (≤2× week/>2× week), socioeconomic status (grades I–II/III [both non‐manual and manual/IV–V], and recent infection [≤3 versus >3 weeks]).

#### Stability Over Time and Association With Cardiovascular Risk Factors and Phenotypes

Next, Pearson correlation was used in both cohorts to assess the long‐term correlations of each biomarker measured 9 to 10 years apart, and to test cross‐sectional bivariate associations between inflammatory biomarkers and a wide range of cardiometabolic risk factors. More detailed analyses linking BMI, waist circumference, and circulating biomarker levels were then tested at ages 15, 24, 32, 37, and 42 years using multivariable linear regression, with results expressed first as z‐score increases in each biomarker per z‐score increase in BMI (after adjustment for waist circumference and other potentially confounding factors), and then repeating with waist circumference as the independent variable. Associations between inflammatory biomarkers and cumulative risk factor burden were next tested by stratifying each risk factor as either high or low risk based on well‐established criteria used in the classification of metabolic syndrome, and multivariable linear regression models were used to test associations between the number of ‘high‐risk’ factors an individual had and circulating levels of both GlycA and CRP. These associations were first carried out unadjusted, and then additionally adjusted for age, sex, and BMI. Next, associations between each inflammatory biomarker and numerous measures of vascular function were tested in both cohorts using multivariable linear regression, with GlycA and CRP again converted to z‐scores to allow comparisons between each.

#### Cardiovascular Risk in Long‐Term Follow‐Up

Finally, baseline levels of GlycA and CRP were used as exposures in modified Poisson regression models with robust error variance in order to calculate risk ratios for hypertension or metabolic syndrome at study follow‐up. Both threshold (quartiles) and continuous (per z‐score change) associations were tested. Four models were created for each inflammatory biomarker: Model 1=unadjusted; Model 2=model 1+adjustments for baseline age, sex, and BMI; Model 3=model 2+adjustments for baseline waist circumference, high‐density lipoprotein cholesterol, triglycerides, glucose, blood pressure, and other inflammatory marker; and Model 4=model 3+adjustments for baseline physical activity levels and socioeconomic status. Multiple imputations (10 imputed data sets) were used to account for missing covariates in statistical models, and details of data missingness can be seen in Table S1. All analyses were conducted using Stata 15.1 (StataCorp LLC, Texas, USA). Where natural log‐transformed data were used as a dependent variable (ie, for CRP), data were back‐transformed to geometric means before reporting. A priori, we planned to draw conclusions based on effect estimates and their 95% CIs, rather than statistical tests using an arbitrary *P* value cutoff of 0.05 (although these are still provided for reference). For example, given 2 effects with the same point estimate—1 with narrow CIs, the other with wider CIs that may even include the null—we described both as showing the same effect. However, 1 is more imprecisely estimated and should be treated with more caution until replicated in a larger sample.

## Results

### Participant Characteristics

Participants in ALSPAC were mean age of 15.4±0.3 years at baseline, 57% female, and followed‐up for an average of 9 years; whereas those in YFS were on average 32.1±5.0 years at baseline, 55% female, and followed‐up for an average of 10 years. All other characteristics are shown in Table 1. No selection bias was apparent in participants included in this study versus the rest of each cohort (Table S2).

**Table 1:** Baseline Characteristics for ALSPAC and YFS cohorts

Variable	ALSPAC			YFS		
	No.	Baseline	Follow‐up	No.	Baseline	Follow‐up
Age, y	1748	15.4±0.3	24.0±0.8	1556	32.1±5.0	42.1±5.0
Sex, % female	1750	57	57	1556	55	55
Height, m	1735	1.69±0.08	1.72±0.09	1547	1.72±0.90	1.72±0.90
Mass, kg	1750	60.6±13.2	73.3±17.9	1547	74.5±16.0	78.9±17.3
BMI, kg/m^2^	1735	20.6 (18.9‐22.7)	23.6 (21.4‐26.8)	1547	24.3 (22.0‐27.3)	25.7 (23.1‐29.1)
Waist circumference, cm	1462	76.3±8.5	80.6±14.9	1530	84.0±12.3	91.7±14.1
SBP, mm Hg	1691	123±11	116±13	1540	122±14	126±15
DBP, mm Hg	1691	66±10	67±8	1540	73±9	79±11
MAP, mm Hg	1691	85±8	83±8	1540	89±10	94±12
LDL‐C, mmol/L	1750	2.1±0.6	2.5±0.8	1538	3.3±0.8	3.3±0.8
HDL‐C, mmol/L	1750	1.3±0.3	1.6±0.4	1555	1.3±0.3	1.3±0.3
Triglycerides, mmol/L	1750	0.8 (0.6–1.0)	0.8 (0.7–1.2)	1556	1.1 (0.8–1.6)	1.1 (0.8–1.6)
Glucose, mmol/L	1750	5.2±0.4	5.3±0.7	1556	5.0±0.8	5.4±1.0
Insulin, µU/mL	1750	9.0 (6.7–11.8)	7.5 (5.3–10.9)	1556	6.0 (5.0–9.0)	7.3 (4.4–11.5)
HOMA2‐IR	1750	1.0 (0.8–1.3)	1.0 (0.7–1.4)	1556	0.8 (0.6–1.2)	1.0 (0.6–1.5)
GlycA, mmol/L	1750	1.21±0.13	1.23±0.17	1556	1.39±0.26	1.60±2.5
CRP, mg/L	1750	0.34 (0.21–0.80)	0.85 (0.39–2.26)	1556	0.71 (0.31–1.67)	0.75 (0.34–1.66)
Physical activity						
CPM	922	472±170	…	…	…	…
PAI	…	…	…	1556	8.86±1.97	…
Socioeconomic status (%)	1456			1522		
I		9.1	…		…	…
II		37.8	…		…	…
III (non‐manual)		40.4	…		…	…
III (manual)		5.4	…		…	…
IV		6.0	…		…	…
V		1.3	…		27.0	…
Higher grade non‐manual		…	…		…	…
Lower grade non‐manual		…	…		43.2	…
Manual		…	…		29.8	…

ALSPAC indicates Avon Longitudinal Study of Parents and Children; BMI, body mass index; CPM, counts per minute; CRP, high‐sensitivity C‐reactive protein; DBP, diastolic blood pressure; GlycA, glycoprotein acetyls; HDL‐C, high‐density lipoprotein cholesterol; HOMA2‐IR, homeostasis model assessment 2 for insulin resistance; LDL‐C, low‐density lipoprotein cholesterol; MAP, mean arterial pressure; PAI, physical activity index; SBP, systolic blood pressure; and YFS, Young Finns Study.

### Stability of GlycA and CRP in Adolescents and Young Adults During 9‐ to 10‐Year Follow‐Up

Levels of both inflammatory biomarkers tended to be lower in adolescence compared with young adulthood (Table 1). Individuals reporting an acute infectious illness in the previous 3 weeks in the ALSPAC cohort had increased levels of both biomarkers compared with those without, with this effect particularly pronounced for CRP (5% versus 121% increase for GlycA and CRP, respectively; *P*<0.001 for both; Figure S1), replicating similar findings which have previously been reported for the YFS cohort.^27^ After excluding individuals reporting illness or those with CRP levels >10 mg/L, repeat measures of GlycA taken 9 to 10 years apart showed a moderate correlation in both cohorts (r=0.36 and 0.41 for ALSPAC and YFS, respectively; *P*<0.001 for both; Figure S2), whereas repeat measures of CRP showed little relationship in ALSPAC (r=0.07) and only a moderate correlation in YFS (r=0.34; Figure S2). Natural log‐transformed data had no impact on GlycA but strengthened the relationship between repeat measures of CRP (r=0.26 and 0.52 for ALSPAC and YFS, respectively; *P*<0.001).

### Associations Between GlycA, CRP, and Adolescent Lifestyle‐Related Risk Factors Linked to the Future Development of CVD

GlycA, but not CRP, was found to be associated with a wide range of lifestyle‐related risk factors in adolescence which have previously been shown to have well‐established links with the future development CVD. These included visceral fat mass, gut‐derived inflammation, low levels of physical activity, smoking status, and low socioeconomic class. Full details of these findings can be seen in Data S2 (Figures S3 through S5).

### Associations Between GlycA, CRP, and Cardiovascular Risk Factors in the Young

**Figure 1:**
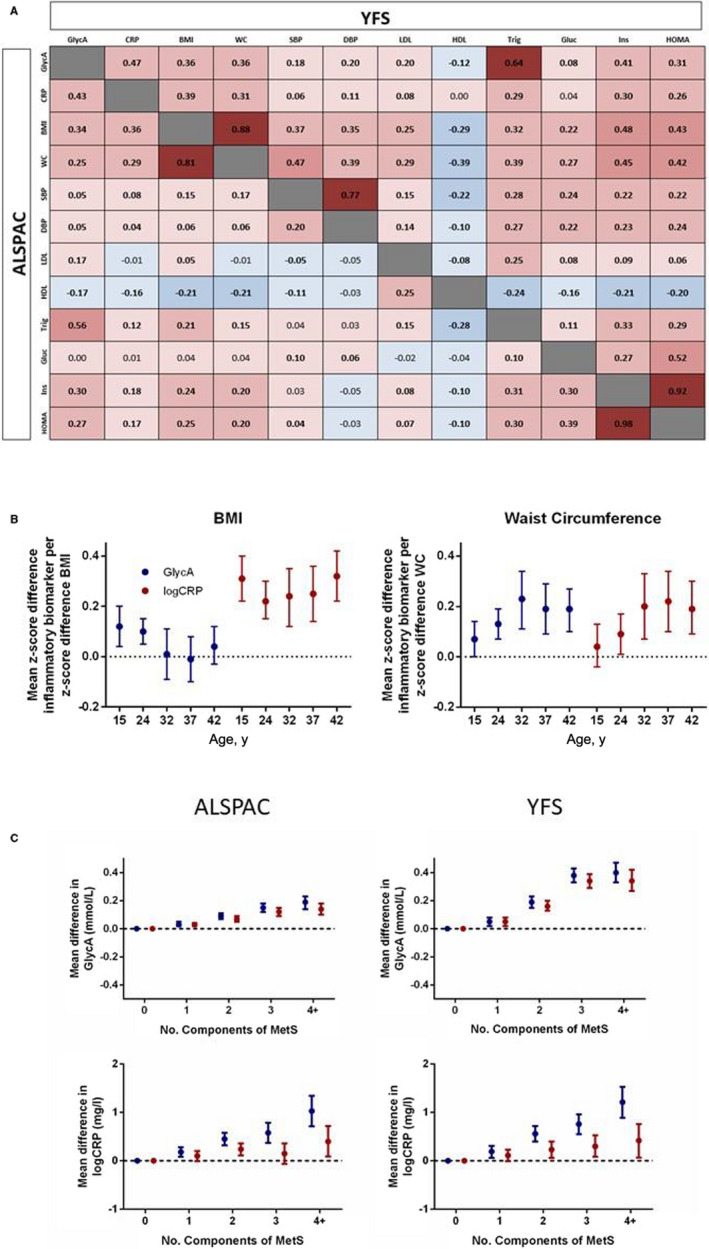
Associations between GlycA, high‐sensitivity CRP, and early markers of cardiovascular risk. (**A**) Bivariate correlations between inflammatory biomarkers and cardiovascular risk factors in both ALSPAC and YFS cohorts. (**B**) Independent associations between BMI, waist circumference, and inflammatory biomarkers during the transition from early to mid‐life. Models adjusted for age, sex, BMI, waist circumference, triglycerides, HDL cholesterol, glucose, other inflammatory biomarker, physical activity levels and socioeconomic status. (**C**) Mean difference in inflammatory biomarker levels per number of adverse cardiovascular risk factors, with results presented unadjusted (blue), and adjusted for age, sex, and body mass index (red). Data in B and C presented as means and 95% CI. ALSPAC indicates Avon Longitudinal Study of Parents and Children; BMI indicates body mass index; CRP, high‐sensitivity C‐reactive protein; DBP, diastolic blood pressure; Gluc indicates glucose; GlycA, glycoprotein acetyls; HDL, high‐density lipoprotein; HOMA, homeostasis model assessment; Ins, insulin; LDL, low‐density lipoprotein; MetS, Metabolic Syndrome; SBP, systolic blood pressure; Trig, triglycerides; WC, waist circumference; and YFS, Young Finns Study.

When assessing univariate associations between each inflammatory biomarker and a range of well‐established cardiovascular risk factors, GlycA in both cohorts was found to associate with body composition, triglycerides, insulin, and Homeostasis Model Assessment 2 for Insulin Resistance most strongly (r=0.25–0.56; Figure 1A). In contrast, CRP in the younger ALSPAC cohort showed little relationship to any risk factor with the exception of body composition (r=0.29–0.36), although in the older YFS cohort it did demonstrate moderate associations with insulin levels and Homeostasis Model Assessment 2 for Insulin Resistance (r=0.26–0.30; Figure 1A). Further investigations of body composition using multivariable regression models demonstrated a positive relationship between waist circumference and both inflammatory biomarkers, with this effect becoming more pronounced during the transition from adolescence to mid‐life (Figure 1B). In contrast, while there was little evidence of any independent relationship between BMI and GlycA at any age, CRP showed consistent positive associations throughout early life (Figure 1B). As such, although both biomarkers increased in a roughly linear fashion with cumulative risk factor burden (Figure 1C), this relationship was attenuated towards the null for CRP in both cohorts after differences in BMI were considered (Figure 1C).

### Relationship Between GlycA, CRP, and Vascular Phenotypes in the Young

**Figure 2:**
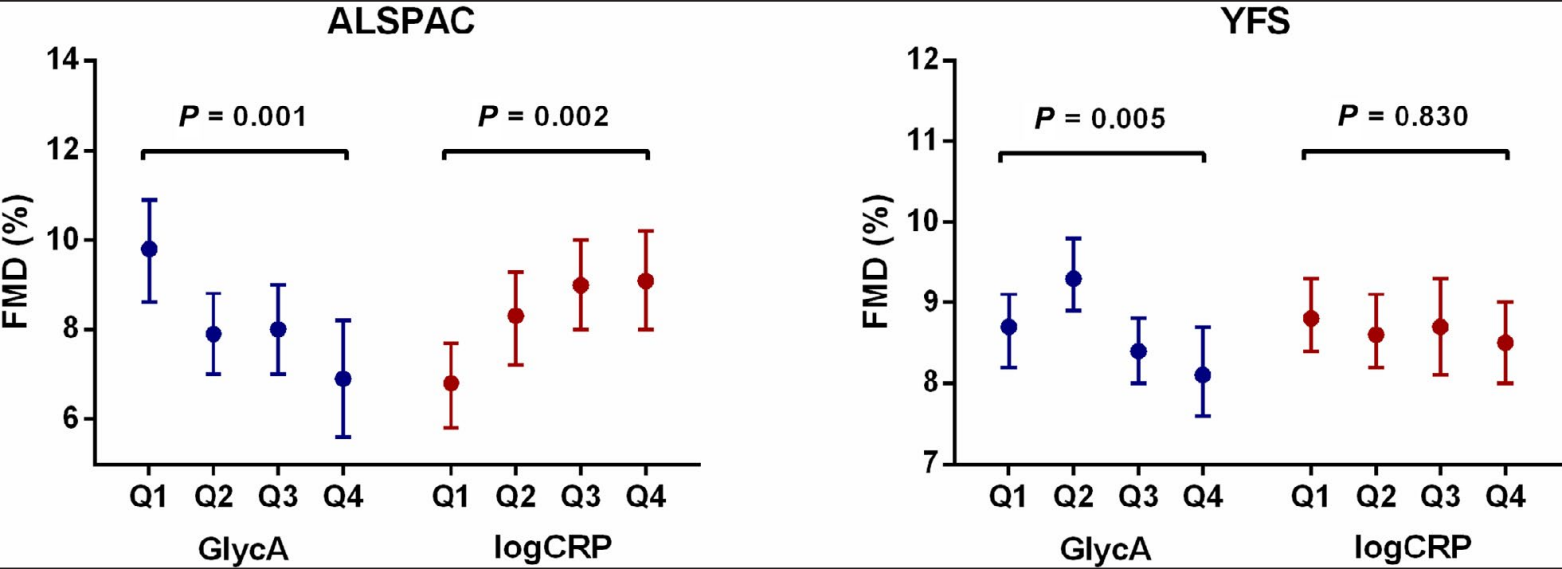
Associations between GlycA, high‐sensitivity CRP, and endothelial dysfunction in the young. Relationship between inflammatory biomarkers and endothelial function assessed via flow‐mediated dilation. Data presented as means and 95% CI adjusted for age, sex, body mass index, triglycerides, high‐density lipoprotein cholesterol, glucose, diastolic blood pressure, and baseline vessel diameter. ALSPAC indicates Avon Longitudinal Study of Parents and Children; CRP indicates C‐reactive protein; FMD, flow‐mediated dilation; GlycA, glycoprotein acetyls; and YFS, Young Finns Study.

Relationships between inflammatory biomarkers and vascular phenotypes were next assessed in the participants with more extensive phenotyping performed at ages 21 (ALSPAC) and 37 years (YFS). In both cohorts, GlycA was found to be associated with impaired endothelial function (mean difference flow‐mediated dilation per z‐score increase GlycA=−1.2 [−1.8, −0.7] and −0.5 [−0.8, −0.2] for ALSPAC and YFS, respectively; *P*<0.003 for both; Table 2 and Figure 2) and higher diastolic blood pressure (mean difference diastolic blood pressure per z‐score increase GlycA=1.2 [0.2, 2.3] and 0.7 [0.0, 1.3], respectively; *P*=0.016 and 0.043; Table 2). In contrast, CRP demonstrated little evidence of a detrimental relationship with any vascular factor in either cohort (Table 2), and in the younger ALSPAC population was in fact found to associate with improved endothelial function (mean difference flow‐mediated dilation per z‐score increase CRP=0.6 [0.2, 1.1]; *P*=0.007; Table 2 and Figure 2).

**Table 2:** Associations Between Inflammatory Biomarkers and Vascular Phenotypes in the ALSPAC and YFS cohorts

Exposure	Outcome	ALSPAC				YFS			
		Unadjusted		Adjusted		Unadjusted		Adjusted	
		Mean difference (95% CI)	*P*	Mean difference (95% CI)	*P*	Mean difference (95% CI)	*P*	Mean difference (95% CI)	*P*
GlycA	SBP (mm Hg)	1.0 (−0.1 to 2.0)	0.067	−0.1 (−1.4 to 1.1)	0.817	2.9 (2.3 to 3.6)	<0.001	−0.2 (−1.1 to 0.7)	0.610
	DBP (mm Hg)	1.8 (1.1 to 2.4)	<0.001	1.2 (0.2 to 2.3)	0.016	2.4 (2.0 to 2.9)	<0.001	0.7 (0.0 to 1.3)	0.043
	MAP (mm Hg)	1.5 (0.8 to 2.1)	<0.001	0.8 (−0.1 to 1.7)	0.098	2.6 (2.1 to 3.1)	<0.001	0.2 (−0.5 to 0.9)	0.510
	IMT (mm)	0.00 (−0.01 to 0.01)	0.843	0.00 (−0.01 to 0.01)	0.745	0.01 (0.00 to 0.01)	0.029	−0.01 (−0.01 to 0.00)	0.034
	FMD (%)	−0.3 (−0.7 to 0.1)	0.179	−1.2 (−1.8 to −0.7)	<0.001	−0.2 (−0.5 to 0.0)	0.040	−0.5 (−0.8 to −0.2)	0.002
logCRP	SBP (mm Hg)	0.9 (−0.1 to 1.9)	0.079	0.1 (−0.9 to 1.1)	0.790	1.9 (1.3 to 2.6)	<0.001	0.7 (−0.1 to 1.4)	0.077
	DBP (mm Hg)	1.2 (0.5 to 1.9)	0.001	−0.2 (−1.0 to 0.6)	0.582	1.9 (1.5 to 2.4)	<0.001	0.6 (0.1 to 1.1)	0.027
	MAP (mm Hg)	1.1 (0.4 to 1.8)	0.001	−0.1 (−0.8 to 0.6)	0.779	1.9 (1.5 to 2.4)	<0.001	0.5 (0.0 to 1.1)	0.065
	IMT (mm)	0.00 (−0.01 to 0.01)	0.359	0.00 (−0.01 to 0.01)	0.901	0.01 (0.00 to 0.02)	<0.001	0.00 (−0.01 to 0.01)	0.994
	FMD (%)	0.5 (0.1 to 0.9)	0.011	0.6 (0.2 to 1.1)	0.007	0.3 (0.1 to 0.5)	0.009	−0.1 (−0.3 to 0.2)	0.613

Mean difference in vascular phenotype per z‐score difference in inflammatory biomarker at ages 21 years (ALSPAC) and 37 years (YFS). Multivariable models adjusted for age, sex, body mass index, waist circumference, triglycerides, high‐density lipoprotein, glucose, and other inflammatory biomarkers. Carotid intima‐media thickness additionally adjusted for SBP and FMD additionally adjusted for baseline vessel diameter. ALSPAC indicates Avon Longitudinal Study of Parents and Children; CRP, C‐reactive protein; DBP, diastolic blood pressure; FMD, flow‐mediated dilation; IMT, carotid intima‐media thickness; MAP, mean arterial pressure; SBP, systolic blood pressure; and YFS, Young Finns Study.

### GlycA, CRP, and the Prediction of Cardiovascular Risk in Up to 10‐Year Follow‐Up

In both ALSPAC and YFS cohorts, multivariable Poisson regression analyses suggested that individuals with increased levels of GlycA at baseline had an increased risk of both hypertension (risk ratio [RR] per z‐score increase in GlycA=1.12 [0.98, 1.28] and 1.08 [1.00, 1.16] for ALSPAC and YFS, respectively; Table 3) and metabolic syndrome (RR per z‐score increase in GlycA=1.32 [1.12, 1.56] and 1.20 [1.09, 1.32] for ALSPAC and YFS, respectively; Table 3) in 9‐ to 10‐year follow‐up. In contrast, CRP was found to have no predictive ability for future hypertension in either cohort (Table 3), and was found to be associated with a lower risk of metabolic syndrome in the younger ALSPAC cohort (RR for z‐score increase in logCRP=0.82 [0.71, 0.94]; Table 3). The use of a summative measure of cardiovascular risk gave similar results to that seen using metabolic syndrome as a dichotomous outcome (Table S3).

**Table 3:** Risk Ratios for Future Hypertension and Metabolic Syndrome in a 9‐ to 10‐Year Follow‐Up of Both ALSPAC and YFS Cohorts

	ALSPAC					YFS				
	Quartile 1	Quartile 2	Quartile 3	Quartile 4	Per 1 SD	Quartile 1	Quartile 2	Quartile 3	Quartile 4	Per 1 SD
Risk ratio (95% CI) for hypertension in 9‐to‐10‐y follow‐up										
GlycA										
Model 1	1 (Ref)	1.26 (0.96 to 1.65)	1.07 (0.81 to 1.42)	1.69 (1.32 to 2.18)^‡^	1.15 (1.06 to 1.25)^†^	1 (Ref)	1.24 (1.03 to 1.51)*	1.46 (1.21 to 1.75)^‡^	1.65 (1.39 to 1.98)^‡^	1.17 (1.12 to 1.23)^‡^
Model 2	1 (Ref)	1.21 (0.92 to 1.58)	0.97 (0.73 to 1.28)	1.35 (1.03 to 1.77)*	1.04 (0.95 to 1.14)	1 (Ref)	1.20 (1.00 to 1.45)	1.34 (1.12 to 1.60)^†^	1.33 (1.11 to 1.59)^†^	1.09 (1.04 to 1.14)^†^
Model 3	1 (Ref)	1.23 (0.94 to 1.62)	1.05 (0.79 to 1.41)	1.56 (1.13 to 2.15)^†^	1.05 (0.94 to 1.17)	1 (Ref)	1.17 (0.97 to 1.40)	1.29 (1.08 to 1.56)^†^	1.15 (0.91 to 1.45)	1.06 (0.99 to 1.13)
Model 4	1 (Ref)	1.23 (0.94 to 1.62)	1.05 (0.78 to 1.40)	1.55 (1.12 to 2.14)^†^	1.12 (0.98 to 1.28)	1 (Ref)	1.19 (0.99 to 1.43)	1.32 (1.09 to 1.58)^†^	1.15 (0.91 to 1.45)	1.08 (1.00 to 1.16)
logCRP										
Model 1	1 (Ref)	1.12 (0.86 to 1.45)	1.28 (0.99 to 1.65)	1.37 (1.07 to 1.76)*	1.13 (1.04 to 1.23)^†^	1 (Ref)	1.08 (0.90 to 1.29)	1.14 (0.96 to 1.36)	1.18 (1.00, 1.40)	1.06 (1.01, 1.12)^†^
Model 2	1 (Ref)	1.02 (0.79 to 1.32)	1.10 (0.86 to 1.42)	0.97 (0.73 to 1.28)	1.00 (0.90 to 1.10)	1 (Ref)	1.02 (0.87 to 1.21)	1.03 (0.87 to 1.21)	1.07 (0.89, 1.28)	1.03 (0.97, 1.09)
Model 3	1 (Ref)	1.02 (0.79 to 1.32)	1.01 (0.78 to 1.31)	0.85 (0.63 to 1.14)	0.99 (0.90 to 1.10)	1 (Ref)	0.92 (0.78 to 1.08)	0.91 (0.77 to 1.08)	0.92 (0.76, 1.11)	1.01 (0.95, 1.07)
Model 4	1 (Ref)	1.02 (0.79 to 1.32)	1.00 (0.78 to 1.31)	0.85 (0.63 to 1.14)	0.95 (0.85 to 1.06)	1 (Ref)	0.91 (0.78 to 1.08)	0.91 (0.77 to 1.07)	0.93 (0.77, 1.12)	0.99 (0.93, 1.05)
Risk ratio (95% CI) for metabolic syndrome (NCEP) in 9‐ to 10‐year follow‐up										
GlycA										
Model 1	1 (Ref)	1.51 (0.97 to 2.36)	1.52 (0.98 to 2.38)	3.41 (2.31 to 5.04)^‡^	1.51 (1.38 to 1.65)^‡^	1 (Ref)	3.14 (1.75 to 5.64)^‡^	5.91 (3.42 to 10.24)^‡^	9.88 (5.81 to 16.82)^‡^	1.63 (1.54 to 1.73)^‡^
Model 2	1 (Ref)	1.39 (0.89 to 2.17)	1.24 (0.80 to 1.94)	2.06 (1.33 to 3.18)^†^	1.25 (1.11 to 1.40)^‡^	1 (Ref)	2.62 (1.47 to 4.68)^†^	4.41 (2.55 to 7.61)^‡^	5.59 (3.25 to 9.61)^‡^	1.38 (1.26 to 1.51)^‡^
Model 3	1 (Ref)	1.29 (0.83 to 2.00)	1.18 (0.75 to 1.85)	1.86 (1.13 to 3.02)*	1.15 (0.99 to 1.32)	1 (Ref)	2.18 (1.23 to 3.87)^†^	3.21 (1.85 to 5.58)^‡^	3.00 (1.69 to 5.32)^‡^	1.20 (1.11 to 1.30)^‡^
Model 4	1 (Ref)	1.29 (0.83 to 2.01)	1.18 (0.75 to 1.86)	1.88 (1.15 to 3.07)*	1.32 (1.12 to 1.56)^†^	1 (Ref)	2.27 (1.28 to 4.01)^†^	3.20 (1.85 to 5.55)^‡^	2.84 (1.60 to 5.03)^‡^	1.20 (1.09 to 1.32)^‡^
logCRP										
Model 1	1 (Ref)	1.17 (0.78 to 1.75)	1.57 (1.08 to 2.30)*	2.12 (1.49 to 3.03)^‡^	1.32 (1.18 to 1.47)^‡^	1 (Ref)	1.88 (1.26 to 2.81)^†^	2.22 (1.51 to 3.27)^‡^	3.21 (2.23 to 4.62)^‡^	1.40 (1.28 to 1.53)^‡^
Model 2	1 (Ref)	0.94 (0.64 to 1.32)	1.13 (0.78 to 1.64)	0.90 (0.60 to 1.35)	0.97 (0.85 to 1.10)	1 (Ref)	1.63 (1.11 to 2.40)^†^	1.54 (1.05 to 2.27)*	1.74 (1.16 to 2.62)^†^	1.14 (1.02 to 1.27)*
Model 3	1 (Ref)	0.92 (0.62 to 1.36)	1.00 (0.69 to 1.48)	0.66 (0.44 to 1.03)	0.91 (0.80 to 1.04)	1 (Ref)	1.25 (0.87 to 1.79)	1.02 (0.70 to 1.48)	0.91 (0.61 to 1.34)	1.02 (0.92 to 1.13)
Model 4	1 (Ref)	0.93 (0.63 to 1.37)	1.02 (0.69 to 1.49)	0.66 (0.43 to 1.01)	0.82 (0.71 to 0.94)^†^	1 (Ref)	1.26 (0.88 to 1.81)	1.02 (0.70 to 1.48)	0.92 (0.62 to 1.37)	0.98 (0.88 to 1.10)

Model 1 = unadjusted; Model 2 = Model 1 + adjustments for baseline age, sex, and body mass index; Model 3 = Model 2 + adjustments for baseline waist circumference, high‐density lipoprotein cholesterol, triglycerides, glucose, blood pressure, and other inflammatory marker; Model 4 = Model 3 + adjustments for baseline physical activity levels and socioeconomic status. ALSPAC indicates Avon Longitudinal Study of Parents and Children; CRP, C‐reactive protein; GlycA, glycoprotein acetyls; NCEP, National Cholesterol Education Program; Ref, Reference group; and YFS, Young Finns Study

* *P*<0.05.

^†^ *P*<0.01.

^‡^ *P*<0.001.

## Discussion

To our knowledge, we provide the first evidence of an association between a novel biomarker of chronic low‐grade inflammation and multiple markers of early cardiovascular risk in 2 extensively phenotyped longitudinal cohorts containing over 3300 young people followed‐up for 9 to 10 years. Our findings show that (1) GlycA is a stable marker of chronic inflammation which potentially lies downstream of different immune‐related pathways to that underlying CRP, (2) that GlycA is associated with a wide‐range of well‐established lifestyle‐related CVD risk factors for cardiovascular disease even at this early age, (3) that GlycA is associated with widespread cardiometabolic and vascular abnormalities from as early as adolescence, and—unlike CRP—these associations do not appear to be confounded by BMI, and (4) that GlycA—but not CRP—predicts surrogate measures of cardiovascular risk up to 10 years in the future.

Inflammation is increasingly recognized as a hallmark for both type 2 diabetes and cardiovascular disease,^28^ and increasing evidence suggests that lifetime rather than contemporary exposure may pose the greatest risk for future disease prevalence and events.^29^ Despite a long‐hypothesized link between chronic inflammation and the early development of adverse cardiovascular risk profiles, studies using the inflammatory biomarker CRP to link these factors in the young have to‐date produced equivocal results.^5^, ^6^, ^7^, ^8^, ^9^, ^10^, ^11^, ^30^, ^31^ In the current study, we therefore sought to compare and contrast associations between CRP and a novel inflammatory biomarker (GlycA) for the presence and future prediction of cardiovascular risk in 2 independent cohorts spanning an age range from adolescence to mid‐adulthood. Our findings support a number of lines of prior evidence suggesting that GlycA may capture different upstream inflammatory pathways to CRP and may therefore be a more suitable measure of low‐grade chronic inflammation in early life.

Firstly, GlycA has previously been shown to be considerably more stable over time than CRP,^27^ with the latter often virtually undetectable in young healthy individuals except in the case of acute infections.^32^ These findings were confirmed in the current study, where repeat measures of GlycA taken up to 10 years apart showed stronger correlations than those seen for CRP. This was particularly evident in the younger adolescent cohort where overall CRP levels were low, with <5% of individuals found to be >3mg/L level commonly used as a threshold for increased CVD risk in older or clinical populations.^33^ As well as being more stable over time, GlycA was also found to be less sensitive to acute infection, increasing on average by only ≈5% in individuals reporting acute illness in the previous 3 weeks, compared with ≈120% for CRP. Taken together, these findings suggest that GlycA and CRP may capture inflammatory burden in a similar manner to that seen for HbA1c and fasting glucose when assessing blood sugar control; with the former more representative of longer‐term exposure to cumulative inflammatory burden, and the latter more sensitive to acute changes which accompany infectious illness.

We next sought to characterize multiple lifestyle and anthropometric factors which may drive inflammation at this early age and associate these with differences in each of the inflammatory biomarkers. In individuals as young as 15 years old, we observed evidence of increased GlycA levels in those with low levels of physical activity, low socioeconomic status, and in those who reported having tried smoking in their early years. These findings suggest the presence of a chronic low‐grade inflammatory burden which may not be detected using CRP, either because of a lack of power arising from its higher variability, or a failure to adequately capture the upstream inflammatory pathways important at this age. They also suggest that even low levels of cigarette use at this young age may have early detrimental effects on cardiovascular health—a phenomenon we have already shown evidence for in the ALSPAC cohort.^34^ In contrast to this aforementioned study, no effect was seen for alcohol intake, although it should be noted that this was relatively rare given the young age. In both cohorts, GlycA was found to be predominantly associated with waist circumference rather than BMI, with this effect becoming more pronounced as individuals transitioned into later life. These findings suggest an important role for abdominal adiposity in the production and release of glycoprotein acetyls into the circulation,^35^ a theory which was strengthened here by the observation of a relationship between visceral fat mass and GlycA in a subset of patients with additional magnetic resonance imaging phenotyping. Furthermore, the additional measurement of LBP/sCD14 ratio in these same individuals highlighted the potential for gut‐associated pathways to act as another possible source of inflammation from the abdominal area. These findings again agree with previous research in which increases in GlycA levels have been found to relate to endotoxemia,^36^ gut dysbiosis,^37^, ^38^ and low fruit and vegetable intake^39^; with the latter relationship seemingly mediated through both the gut microbiome and circulating lymphocyte levels. In contrast, and again in agreement with previous research,^5^, ^9^, ^30^ elevated CRP levels appeared to be driven predominantly by BMI and subcutaneous fat mass at a young age, although waist circumference did appear to play a greater role as individuals moved towards middle‐age. This close relationship between BMI and CRP is well‐established in the literature and has been suggested to be a potentially confounding factor in many studies relating low‐grade inflammation to early changes in cardiovascular risk profiles. This theory appears to be supported here, with CRP found to relate to few risk factors except for BMI at a young age, and for any associations between CRP and cumulative risk factor burden found to attenuate towards null once accounting for differences in BMI.

Similar findings were observed when comparing each of the measured biomarkers with a range of vascular phenotypes commonly used as subclinical markers of increased cardiovascular risk. Even after adjustments for BMI and a wide range of other potentially confounding factors, GlycA was found to consistently associate with impaired endothelial function and increased diastolic blood pressure both in adolescence and young adulthood, suggesting a link between chronic inflammatory processes and early systemic vascular dysfunction. While similar findings between GlycA and vascular dysfunction have previously been reported in patients with psoriasis,^40^ results presented here are the first to show similar associations in young and otherwise healthy individuals free from established inflammatory disease. While CRP did show some evidence of a modest relationship with increased blood pressure, these effects were once again attenuated towards null once accounting for potential confounders such as BMI. Interestingly, there was some evidence of an improvement in endothelial function in younger individuals with higher levels of CRP, a finding which has also been demonstrated in previous studies in relationship to other vascular measures such as carotid intima‐media thickness.^12^, ^41^ While we cannot explain this association, we have previously shown that obesity is paradoxically associated with improved endothelial function in the young, most likely because of the presence of a chronic hyperemic state accompanying higher body mass. As such, associations between CRP and endothelial function may also be affected by residual confounding from BMI at this age, although this warrants further investigation.

Given the demonstrated link between GlycA and numerous cardiovascular risk factors in cross‐sectional analyses of both cohorts, we next assessed the ability of each biomarker to predict future CVD risk in long‐term follow‐up. In the absence of hard disease outcomes at this age, we chose to represent this increased risk using subclinical measures with well‐established links to future CVD—namely hypertension and metabolic syndrome.^42^, ^43^ A number of previous studies in older clinical populations have demonstrated the ability for GlycA to predict both CVD and cardiometabolic (type 2 diabetes) disease independently of CRP in long‐term follow‐up.^15^, ^16^, ^44^, ^45^ Here, we found GlycA to be associated with an increased risk of both hypertension and metabolic syndrome in both cohorts 9 to 10 years after baseline measures. Notably, while CRP was also associated with increased risk in age‐ and sex‐adjusted models, this relationship not only disappeared, but in the case of metabolic syndrome in the younger ALSPAC cohort, was again reversed once other potential confounding factors such as BMI were considered. Why this occurs is not clear, and—like the association with flow‐mediated dilation—warrants further investigation.

This study is not without its limitations. Firstly, as with all cohort studies, it is not possible to definitively determine the direction of relationships between observed associations, and further interventional or genetic studies are required to answer this question. However, the finding in both cohorts of an increased 9‐to‐10‐year risk of both hypertension and metabolic syndrome in individuals with elevated levels of GlycA provides some suggestion of a detrimental impact of inflammation on future CVD risk. Second, it was not possible to discern whether an increased level of GlycA represents a causal risk factor for disease itself or—like CRP—is more likely simply a biomarker increasing in response to other upstream causal pathways.^1^, ^46^ Given the heterogeneous nature of the acute phase proteins which constitute the GlycA NMR signal, this will prove difficult to test, as 2 individuals with the same GlycA level may have different concentrations and/or glycosylation patterns in underlying proteins. However, as each of these proteins in isolation are generally considered to possess anti‐inflammatory properties, it is reasonable to assume that elevated GlycA levels may represent an ongoing attempt at a protective response to underlying chronic inflammatory burden,^47^ and that interventions targeting pathways upstream are likely to offer the best opportunity for risk reduction. Third, although LBP and sCD14 are commonly used markers of gut‐derived inflammation, we recognize that they are not specific to lipopolysaccharide and may therefore capture other sources of inflammation. Lastly, it has been suggested that the measurement of GlycA by NMR may be liable to confounding by high triglyceride levels. As such, we report only multivariable models containing adjustments for serum triglycerides as our outcomes of interest.

In conclusion, we provide the first evidence of an association between circulating glycoprotein acetyls and adverse cardiovascular risk profiles in 2 separate cohorts spanning an age range from adolescence to mid‐life. GlycA may capture different upstream inflammatory pathways underlying cardiovascular risk at this age and may be a more sensitive inflammatory biomarker than CRP for detecting the emergence of early cardiovascular risk in the young.

## Sources of Funding

The UK Medical Research Council and Wellcome (Grant ref: 217065/Z/19/Z) and the University of Bristol provide core support for ALSPAC. This publication is the work of the authors and STC will serve as guarantor for the contents of this paper. This research was specifically funded through grants from the British Heart Foundation (PG/18/45/33814; CS/15/6/31468), Wellcome Trust and MRC (076467/Z/05/Z), and NIH (R01 DK077659). The Young Finns Study has been financially supported by the Academy of Finland: grants 322098, 286284, 134309 (Eye), 126925, 121584, 124282, 129378 (Salve), 117787 (Gendi), and 41071 (Skidi); the Social Insurance Institution of Finland; Competitive State Research Financing of the Expert Responsibility area of Kuopio, Tampere and Turku University Hospitals (grant X51001); Juho Vainio Foundation; Paavo Nurmi Foundation; Finnish Foundation for Cardiovascular Research; Finnish Cultural Foundation; The Sigrid Juselius Foundation; Tampere Tuberculosis Foundation; Emil Aaltonen Foundation; Yrjö Jahnsson Foundation; Signe and Ane Gyllenberg Foundation; Diabetes Research Foundation of Finnish Diabetes Association; EU Horizon 2020 (grant 755320 for TAXINOMISIS and grant 848146 for To Aition); European Research Council (grant 742927 for MULTIEPIGEN project); Tampere University Hospital Supporting Foundation and the Finnish Society of Clinical Chemistry. MAK has a research grant from the Sigrid Juselius Foundation, Finland.

## Disclosures

None.

## Supporting information

Data S1
Tables S1–S3
Figures S1–S5
