# Supplementary material for: Glycoprotein Acetyls: A Novel Inflammatory Biomarker of Early Cardiovascular Risk in the Young
Source: J Am Heart Assoc. 2022 Feb 12;11(4):e024380. doi: 10.1161/JAHA.121.024380 (PMC9245818; doi:10.1161/JAHA.121.024380)
Supplement: Supplementary file 1 — Data S1 Tables S1–S3 Figures S1–S5 [file JAH3-11-e024380-s001.pdf]

SUPPLEMENTAL MATERIAL

DATA S1. EXPANDED METHODS

Detailed Cohort Descriptions

ALSPAC

Pregnant women resident in Avon, UK with expected dates of delivery 1st April 1991 to 31st December 1992 were invited to take part in the study. The initial number of pregnancies enrolled is 14,541 (for these at least one questionnaire has been returned or a "Children in Focus" clinic had been attended by 19/07/99). Of these initial pregnancies, there was a total of 14,676 foetuses, resulting in 14,062 live births and 13,988 children who were alive at 1 year of age. When the oldest children were approximately 7 years of age, an attempt was made to bolster the initial sample with eligible cases who had failed to join the study originally. As a result, when considering variables collected from the age of seven onwards (and potentially abstracted from obstetric notes) there are data available for more than the 14,541 pregnancies mentioned above. The number of new pregnancies not in the initial sample (known as Phase I enrolment) that are currently represented on the built files and reflecting enrolment status at the age of 24 is 913 (456, 262 and 195 recruited during Phases II, III and IV respectively), resulting in an additional 913 children being enrolled. The phases of enrolment are described in more detail in the cohort profile paper and its update (see footnote 4 below). The total sample size for analyses using any data collected after the age of seven is therefore 15,454 pregnancies, resulting in 15,589 foetuses. Of these 14,901 were alive at 1 year of age. A 10% sample of the ALSPAC cohort, known as the Children in Focus (CiF) group, attended clinics at the University of Bristol at various time intervals between 4 to 61 months of age. The CiF group were chosen at random from the last 6 months of ALSPAC births (1432 families attended at least one clinic). Excluded were those mothers who had moved out of the area or were lost to follow-up, and those partaking in another study of infant development in Avon. Full details of the ALSPAC cohort and study design are available at the ALSPAC website (http://www.alspac.bris.ac.uk). This website also contains details of all data that is available through a fully searchable data dictionary and online variables search tool (http://www.bris.ac.uk/alspac/researchers/data-access/data-dictionary). Study data in the ALSPAC@24 clinic were collected and managed using REDCap electronic data capture tools hosted at University of Bristol. REDCap (Research Electronic Data Capture) is a secure, web-based software platform designed to support data capture for research studies, providing 1) an intuitive interface for validated data capture; 2) audit trails for tracking data manipulation and export procedures; 3) automated export procedures for seamless data downloads to common statistical packages; and 4) procedures for data integration and interoperability with external sources.

YFS

Further details on the YFS cohort and study design can be found on the YFS website (https://youngfinnsstudy.utu.fi/). In 1980, 4320 children aged 3, 6, 9, 12, 15, and 18 years were randomly chosen from the national population register of five areas and invited to participate in the study. Of those invited, 3596 children participated in the first cross-sectional survey. Since then, follow-up studies have been conducted in 1983, 1986, 1989, 1992, 2001, 2007, and 2011. The entire cohort was invited to participate in follow-up studies in 1983, 1986, 2001, 2007, and 2011 when 2991, 2799, 2620, 2243, and 2115 subjects participated, respectively. In 1989 and 1992, physical examination and blood tests were gathered from a subsection of the cohort. In addition, during these two follow-ups, background information questionnaire was gathered from the entire cohort and

ultrasound imaging and adulthood cardiovascular risk factors were collected. The datasets presented in this article are not readily available because YFS is an ongoing follow-up study and the datasets are not anonymised, and the GDPR prevents public sharing of the data. Instead, pseudonymised datasets are possible to share on request, and requires a data sharing agreement between the parties. Requests to access the datasets should be directed to YFS study coordinators.

Classification of Hypertension and Metabolic Syndrome in ALSPAC and YFS Cohorts

Hypertension in the ALSPAC cohort was classed as age- and sex-specific systolic or diastolic blood pressure ≥80th percentile due to the young age of the participants and their transition through adolescence during the course of follow-up. In YFS, a cut-off of ≥130mmHg (systolic), ≥85mmHg (diastolic), or evidence of blood pressure treatment was used. MetS in ALSPAC was characterised using a modified version of the National Cholesterol Education Programme (NCEP) guidelines. In this version, a participant was classified as having MetS if they had any three of the following five age- and sex-specific components: elevated waist circumference ≥80th percentile, systolic or diastolic blood pressure, triglycerides ≥80th percentile, glucose ≥80th percentile, or HDL-C ≤20th percentile. In the older YFS cohort, standard NCEP guidelines were used: waist >102cm in men and >88cm in women, serum triglycerides ≥1.695mmol/l (150mg/dl), HDL cholesterol <1.036mmol/l (40mg/dl) in men and 1.295mmol/l (50mg/dl) in women, blood pressure ≥130mmHg (systolic) or ≥85mmHg (diastolic) or treated, and plasma glucose ≥5.6mmol/l (110mg/dl). To complement the dichotomous definition, a continuous MetS risk score was also calculated to represent summative cardiometabolic risk factor burden. For the continuous score, the components were first standardized (z-scored) for age and sex (HDL-C was multiplied by −1) and then summed by age. As a representation of blood pressure, the mean of the systolic and diastolic blood pressure values was used.

Detailed Vascular Imaging Description

Carotid Intima-Media Thickness (cIMT)

ALSPAC – Right and left common carotid arteries were imaged using an ultrasound machine (CardioHealth Panasonic) and 13.5MHz linear array broadband transducer (probe centre frequency 9.0MHz). Optimal end-diastolic images of cIMT were measured 1cm proximal to the carotid bifurification. Both the right and left common carotid artery scans were imaged longitudinally to include the common carotid artery and the carotid bifurcation, and automated measurements of cIMT were collected from the far wall of the vessel. In statistical analyses, the mean of the left and right carotid arteries was used as a continuous outcome variable.

YFS – cIMT was measured using Sequoia 512 ultrasound mainframes (Acuson, CA) with 13.0 MHz linear array transducers. Carotid IMT was measured from the carotid artery bifurcation (bulb region) and evident plaque lesions were documented (defined as distinct area of the carotid vessel wall protruding into the lumen >50% of the adjacent intima-media layer). All plaques were observed in the carotid bulb. The digitally stored scans were manually analyzed by one reader blinded to subjects' details. The between-visit coefficient of variation of IMT measurements was 6.4% and the intra-observer coefficient of variation was 3.4% in our laboratory. A continuous measure of cIMT was measured on the posterior (far) wall of the left common carotid artery and three measurements were performed to derive mean and maximal common carotid IMT. In statistical analyses, the mean value was used as a continuous outcome variable.

Flow Mediated Dilation (FMD)

ALPSAC – FMD was measured with the right arm extended perpendicular to the body and secured in a custom-built clamp. Duplex Doppler ultrasound (Aloka 5500, Hitachi, Japan) was used to image the brachial arterial diameter for 1min at rest, during 5mins of pneumatic cuff inflation (200mmHg) at the level of the forearm, and then again for 5 mins post-occlusion. Automated software (Brachial Analyzer 5.0, MIA, US) was used to continuously track changes in the arterial diameter over this time, and FMD was reported as peak % change following cuff deflation.

YFS **-** Brachial FMD was examined by measuring the left brachial artery diameter both at rest and during reactive hyperemia. The increased blood flow was induced by inflating a pneumatic tourniquet placed around the forearm to a pressure of 250mmHg for 4.5 minutes and then deflating the tourniquet. Measurement of arterial diameter was performed at end-diastole at fixed distance from an anatomic marker at rest and 40, 60 and 80 seconds after cuff release. The maximum vessel diameter during dilatation was expressed as the percentage relative to resting scan.

DATA S2. EXPANDED RESULTS

Associations between GlycA, CRP, and adolescent lifestyle-related risk factors linked to the future development of CVD

DEXA measures of fat and lean mass collected at age 15 revealed that while GlycA levels in adolescence were positively associated with levels of absolute fat mass, increased levels of lean mass appeared to attenuate this association at any level of adiposity ($p < 0.001$; Figure S3A). This attenuating effect of increased lean mass was also seen for CRP at low and moderate levels of adiposity, but was lost when fat mass was high, resulting in CRP levels in those with a high fat mass that was 3.5-4x that seen in low and moderate fat groups regardless of underlying lean mass (Figure S3A). Multivariable analyses using MRI-derived measures of fat distribution showed a positive relationship between visceral fat mass and GlycA (mean z-score difference 0.13 [0.07, 0.25; p = 0.038), and between subcutaneous fat mass and CRP (mean z-score difference 0.21 [0.05, 0.37]; p = 0.008; Figure S3B). No relationship was observed between LPS levels and either biomarker, but GlycA was found to associate with the ratio of circulating LBP/sCD14 (mean difference 3.6 [0.4, 6.8]; p = 0.027; Figure S3C).

In further multivariable analyses assessing physical activity, we found increased levels of physical activity to be associated with lower levels of circulating GlycA (p = 0.002), but not CRP (p = 0.170; Figure S4A). When additionally categorising by BMI, increased physical activity levels were generally associated with lower levels of both biomarkers regardless of obesity status, with this effect particularly pronounced for CRP levels in those belonging to the highest BMI tertile (Figure S4B).

Both GlycA and CRP were elevated in adolescents who reported a previous history of trying smoking compared to those who did not (p = 0.009 and 0.035, respectively; Figure S5A), whereas alcohol intake (<=2 drinks/week vs. >2 drinks/week) showed little evidence of a relationship to either biomarker (p = 0.956 for both; Figure S5B). Finally, only GlycA was found to be increased in those from lower socioeconomic classes (mean difference 0.04 [0.01, 0.07] mmol/L for grades IV-V vs groups I-II; p = 0.003; Figure S5C).

Table S1: Data missingness for exposure variables in ALSPAC and YFS

Exposure Variable	Missing Observations	
	**ALSPAC**	**YFS**
Age	2 (0%)	0 (0%)
Sex	0 (0%)	0 (0%)
Height	15 (1%)	9 (1%)
Mass	0 (0%)	9 (1%)
BMI	15 (1%)	9 (1%)
Waist Circumference	288 (16%)	26 (2%)
SBP	59 (3%)	16 (1%)
DBP	59 (3%)	16 (1%)
MAP	59 (3%)	16 (1%)
LDL-c	0 (0%)	18 (1%)
HDL-c	0 (0%)	1 (0%)
Triglycerides	0 (0%)	0 (0%)
Glucose	0 (0%)	0 (0%)
Insulin	0 (0%)	0 (0%)
HOMA2-IR	0 (0%)	0 (0%)
GlycA	0 (0%)	0 (0%)
CRP	0 (0%)	0 (0%)
MetS	0 (0%)	0 (0%)
Physical Activity	828 (47%)	0 (0%)
Social Class	294 (17%)	34 (2%)

BMI, body mass index; SBP, systolic blood pressure; DBP, diastolic blood pressure; MAP, mean arterial pressure; LDL-c, low density lipoprotein cholesterol; HDL-c, high-density lipoprotein cholesterol; HOMA2-IR, homeostasis model assessment for insulin resistance; GlycA, glycoprotein acetyls; CRP, high-sensitivity C-reactive protein; MetS, metabolic syndrome (NCEP criteria)

Table S2: Comparison of cohort characteristics in participants included in analysis vs those excluded

	ALSPAC		YFS	
**Variable**	**Participants with NCEP outcome (included in analysis)**	**Participants without NCEP outcome (excluded from analysis)**	**Participants with NCEP outcome (included in analysis)**	**Participants without NCEP outcome (excluded from analysis)**
Age (years)	15.4 ± 0.3	15.5 ± 0.4	32.1 ± 5.0	30.9 ± 1.9
Sex (% female)	57	49	55	52
Height (m)	1.69 ± 0.08	1.69 ± 0.08	1.72 ± 0.90	1.72 ± 0.92
Mass (kg)	60.6 ± 13.2	62.0 ± 12.4	74.5 ± 16.0	74.7 ± 16.0
BMI (kg/m^2)	20.6 (18.9, 22.7)	20.8 (19.1, 23.2)	24.3 (22.0, 27.3)	24.5 (22.0, 27.6)
Waist Circumference (cm)	76.3 ± 8.5	77.1 ± 9.3	84.0 ± 12.3	84.3 ± 12.3
SBP (mmHg)	123 ± 11	122 ± 12	122 ± 14	123 ± 15
DBP (mmHg)	66 ± 10	67 ± 10	73 ± 9	73 ± 9
MAP (mmHg)	85 ± 8	85 ± 8	89 ± 10	89 ± 10
LDL-c (mmol/l)	2.1 ± 0.6	2.1 ± 0.6	3.3 ± 0.8	3.2 ± 0.9
HDL-c (mmol/l)	1.3 ± 0.3	1.3 ± 0.3	1.3 ± 0.3	1.3 ± 0.3
Triglycerides (mmol/l)	0.8 (0.6, 1.0)	0.8 (0.6, 1.0)	1.1 (0.8, 1.6)	1.1 (0.8, 1.6)
Glucose (mmol/l)	5.2 ± 0.4	5.2 ± 0.4	5.0 ± 0.8	5.1 ± 0.1
Insulin (µU/ml)	9.0 (6.7, 11.8)	9.2 (6.9, 12.7)	6.0 (5.0, 9.0)	7.0 (4.0, 9.0)
HOMA2-IR	1.0 (0.8, 1.3)	1.0 (0.8, 1.4)	0.8 (0.6, 1.2)	1.1 ± 0.9
GlycA (mmol/L)	1.21 ± 0.13	1.22 ± 0.13	1.39 ± 0.26	1.41 ± 0.27
CRP (mg/l)	0.34 (0.21, 0.80)	0.39 (0.22, 0.90)	0.71 (0.31, 1.67)	0.80 (0.34, 1.93)
Physical Activity				
(CPM)	472 ± 170	490 ± 190	-	-
(PAI)	-		8.86 ± 1.97	8.85 ± 1.94
Socioeconomic Status (%)				
- I	9.1	6.2	-	-
- II	37.8	33.6	-	-
- III (non-manual)	40.4	41.8	-	-
- III (manual)	5.4	7.9	-	-
- IV	6.0	9.2	-	-
- V	1.3	1.4	-	-
- Higher Grade Non-Manual	-	-	27.0	35.2
- Lower Grade Non-Manual	-	-	43.2	42.6
- Manual	-	-	29.8	22.2

BMI, body mass index; SBP, systolic blood pressure; DBP, diastolic blood pressure; MAP, mean arterial pressure; LDL-c, low density lipoprotein cholesterol; HDL-c, high-density lipoprotein cholesterol; HOMA2-IR, homeostasis model assessment for insulin resistance; GlycA, glycoprotein acetyls; CRP, high-sensitivity C-reactive protein; CPM, counts per minute; PAI, physical activity index

Table S3: Mean increase in summative cardiometabolic risk z-score per quartile and 1-SD increase in inflammatory biomarkers

**ALSPAC**					
	Quartile 1	Quartile 2	Quartile 3	Quartile 4	Per 1 SD
GlycA					
Model 1	1 (Ref)	0.54 (0.17, 0.90)†	0.61 (0.24, 0.97)†	1.78 (1.40, 2.15)†	0.69 (0.55, 0.82)†
Model 2	1 (Ref)	0.39 (0.05, 0.73)*	0.25 (-0.10, 0.59)	1.01 (0.64, 1.38)†	0.37 (0.23, 0.50)†
Model 3	1 (Ref)	0.20 (-0.13, 0.53)	0.03 (-0.31, 0.38)	0.47 (0.05, 0.90)*	0.20 (0.03, 0.38)*
Model 4	1 (Ref)	0.20 (-0.13, 0.53)	0.03 (-0.32, 0.37)	0.48 (0.05, 0.90)*	0.21 (0.03, 0.38)*
logCRP					
Model 1	1 (Ref)	0.23 (-0.12, 0.59)	0.52 (0.15, 0.89)#	1.15 (0.77, 1.51)†	0.41 (0.28, 0.54)†
Model 2	1 (Ref)	-0.12 (-0.46, 0.21)	-0.06 (-0.41, 0.28)	-0.05 (-0.41, 0.32)	-0.03 (-0.16, 0.10)
Model 3	1 (Ref)	-0.15 (-0.47, 0.17)	-0.16 (-0.50, 0.17)	-0.38 (-0.74, -0.01)*	-0.18 (-0.32, -0.04)*
Model 4	1 (Ref)	-0.15 (-0.47, 0.17)	-0.15 (-0.49, 0.18)	-0.38 (-0.75, -0.02)*	-0.19 (-0.32, -0.05)#
**YFS**					
	Quartile 1	Quartile 2	Quartile 3	Quartile 4	Per 1 SD
GlycA					
Model 1	1 (Ref)	1.16 (0.75, 1.57)†	2.12 (1.71, 2.53)†	3.16 (2.75, 3.57)†	1.19 (1.04, 1.33)†
Model 2	1 (Ref)	0.59 (0.24, 0.95)#	1.21 (0.84, 1.57)†	1.58 (1.20, 1.96)†	0.66 (0.52, 0.79)†
Model 3	1 (Ref)	0.41 (0.09, 0.73)*	0.67 (0.32, 1.02)#	0.44 (0.03, 0.85)*	0.20 (0.04, 0.36)#
Model 4	1 (Ref)	0.46 (0.15, 0.78)#	0.68 (0.33, 1.02)†	0.39 (-0.02, 0.80)	0.18 (0.02, 0.34)*
logCRP					
Model 1	1 (Ref)	0.79 (0.37, 1.22)#	1.35 (0.93, 1.78)†	2.16 (1.73, 2.59)†	0.79 (0.63, 0.94)†
Model 2	1 (Ref)	0.29 (-0.07, 0.66)	0.48 (0.11, 0.84)*	0.41 (0.01, 0.81)*	0.14 (0.00, 0.28)
Model 3	1 (Ref)	0.12 (-0.20, 0.44)	0.27 (-0.06, 0.60)	-0.07 (-0.44, 0.29)	-0.01 (-0.14, 0.12)
Model 4	1 (Ref)	0.13 (-0.19, 0.44)	0.27 (-0.06, 0.59)	-0.04 (-0.41, 0.32)	0.00 (-0.13, 0.13)

Model 1 = unadjusted; Model 2 = model 1 + adjustments for baseline age, sex, and BMI; Model 3 = model 2 + adjustments for baseline waist circumference, HDL, triglycerides, glucose, blood pressure, and other inflammatory marker; Model 4 = model 3 + adjustments for baseline physical activity levels and socioeconomic status. * $p < 0.05$ # $p < 0.01$ † $p < 0.001$

Figure S1: Inflammatory biomarkers levels in ALSPAC participants reporting acute illness in the previous three weeks

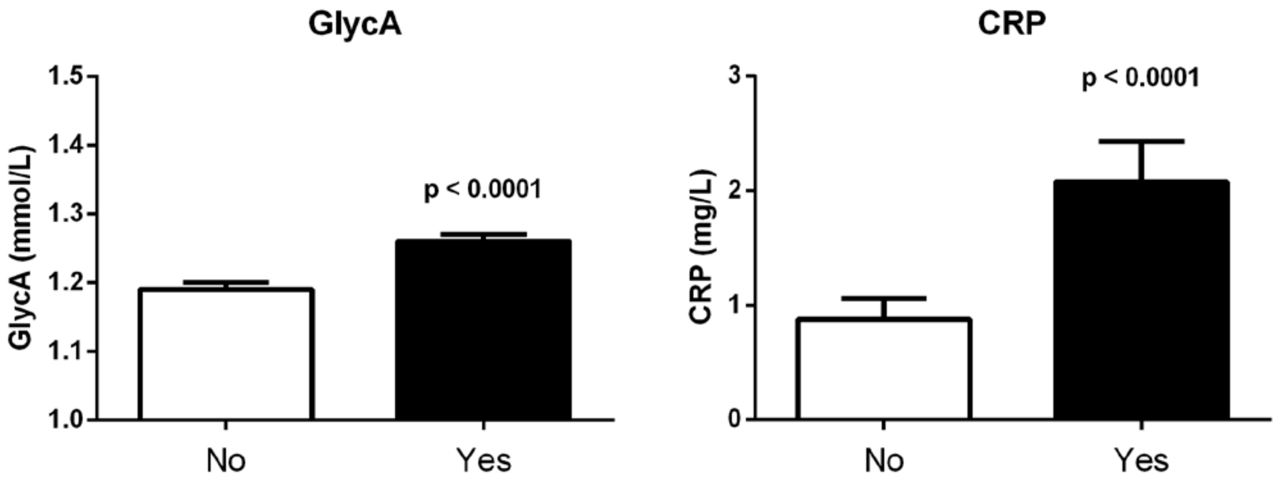

Figure S2: Repeated measures of GlycA and CRP in the same individuals measured up to 9-10 years apart in ALSPAC and YFS cohorts.

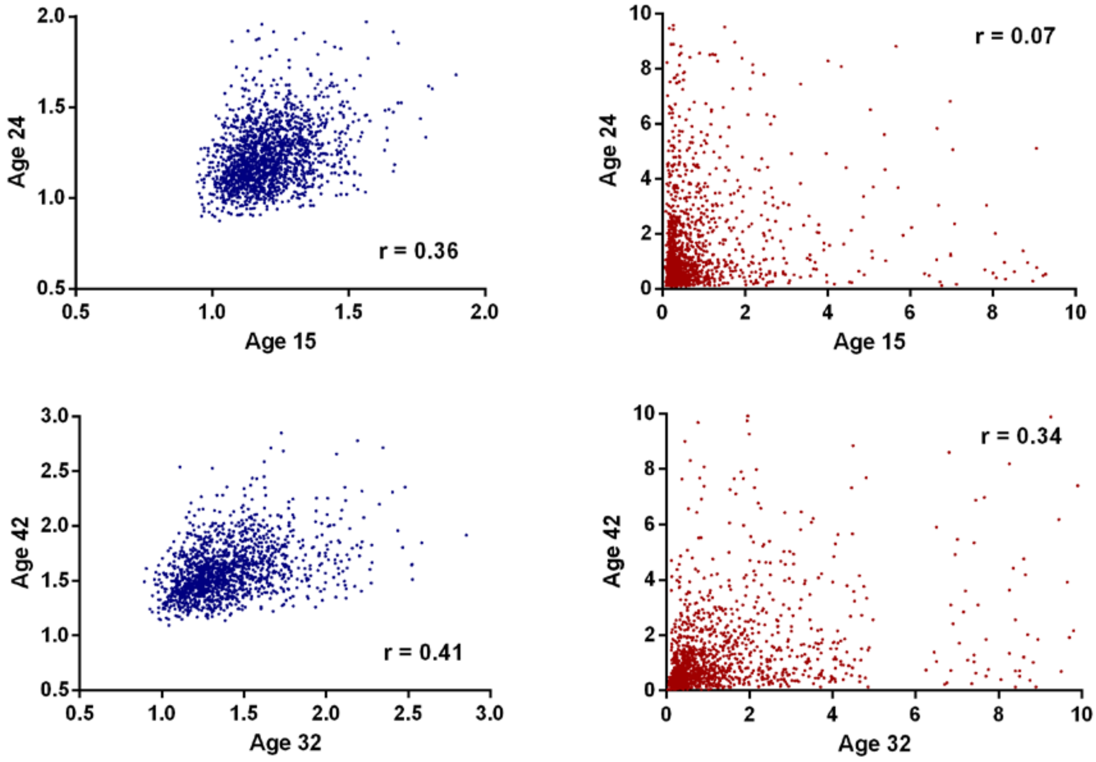

Figure S3: Individual associations of GlycA and CRP with A) fat mass and fat-free mass measured by DEXA, B) fat distribution measured by MRI, and C) LBP/sCD14 ratio

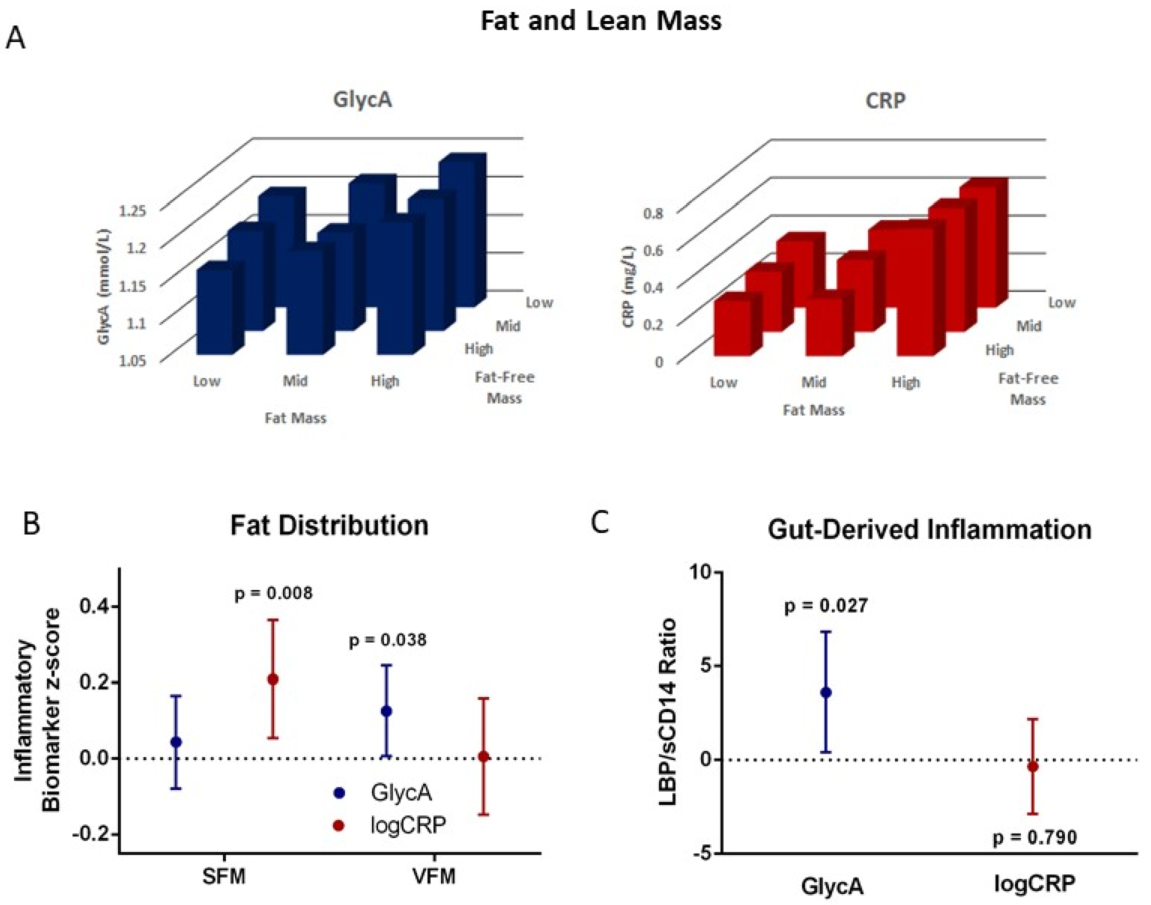

Data in A collected at age 15 in ALSPAC cohort and B and C collected in a subset of same cohort at age 21. All models adjusted for age, sex, triglycerides, HDL, glucose, diastolic blood pressure, physical activity and socioeconomic status. Data in A presented as means and in B as means and 95% CI. Results for CRP are displayed as geometric means.

Figure S4: Individual associations of GlycA and CRP with A) physical activity, and B) BMI and physical activity

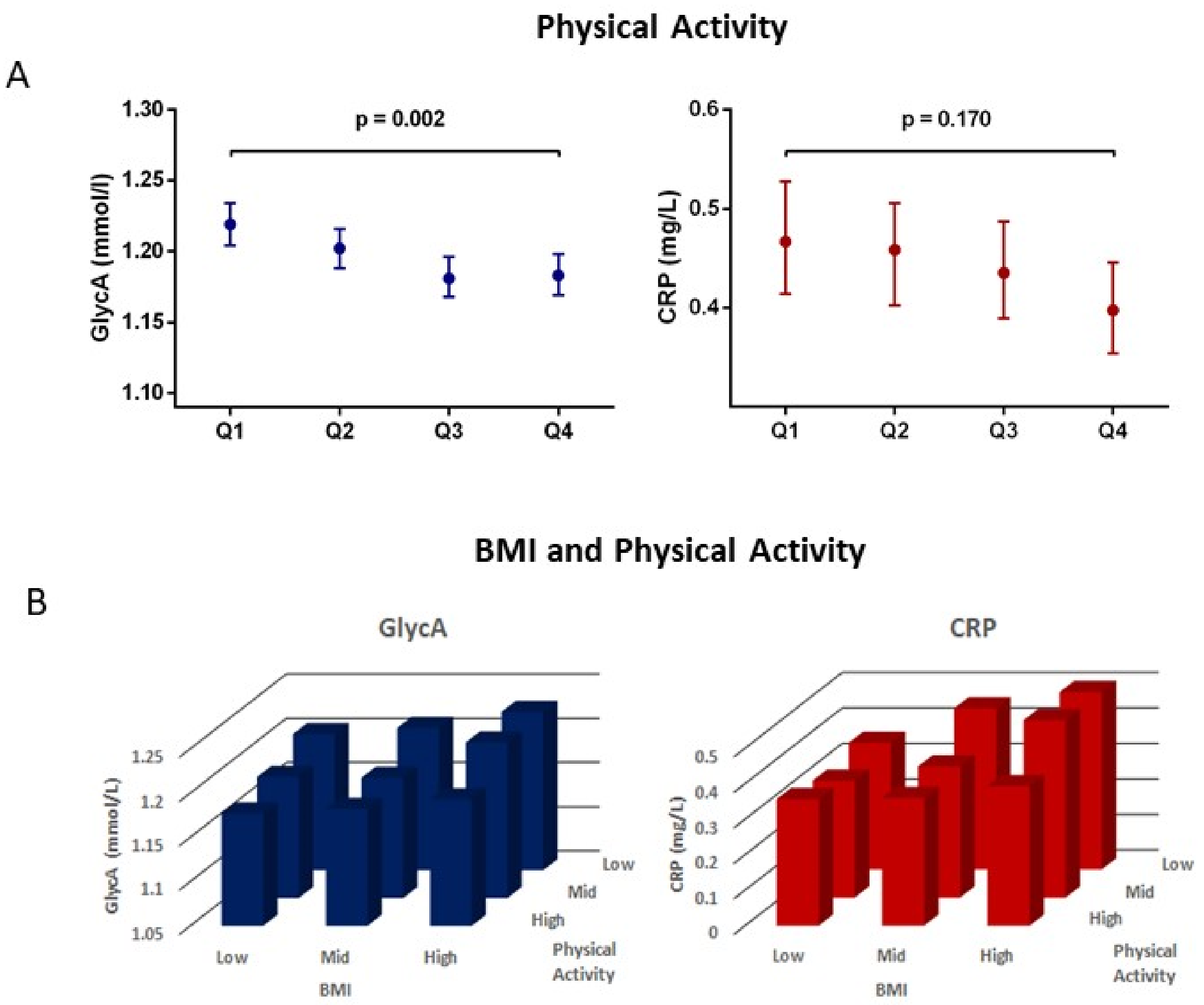

All data collected at age 15 in ALSPAC cohort. All models adjusted for age, sex, BMI (except where exposure of interest) triglycerides, HDL, glucose, diastolic blood pressure, physical activity and socioeconomic status. Data in A presented as means and 95% CI and data in B as means. Results for CRP are displayed as geometric means.

Figure S5: Individual associations of GlycA and CRP with A) smoking, B) alcohol intake, and C) socioeconomic status

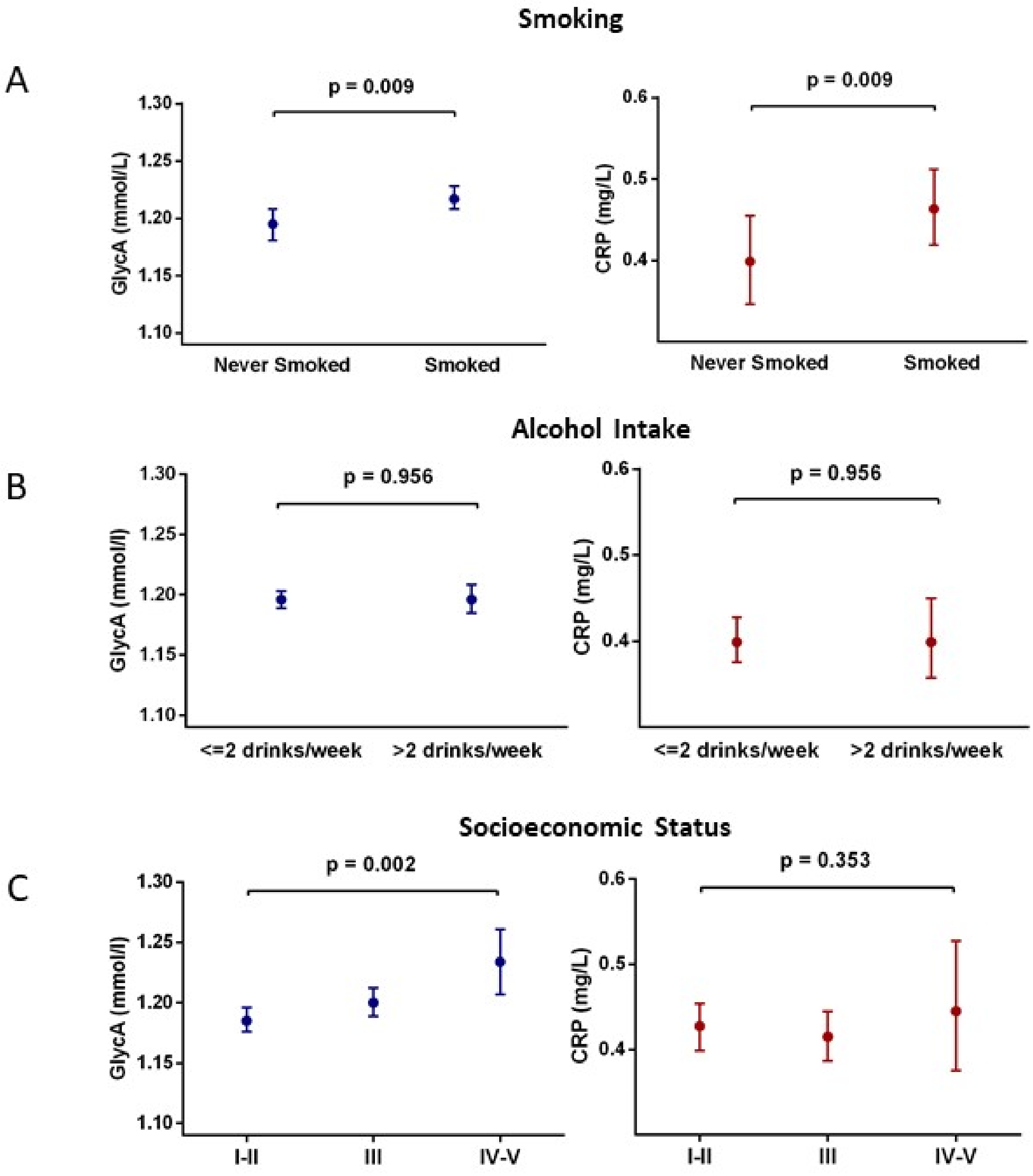

All data collected at age 15 in ALSPAC cohort. All models adjusted for age, sex, BMI and waist circumference, triglycerides, HDL, glucose, diastolic blood pressure, and physical activity and socioeconomic status (except where exposures of interest). All data presented as means and 95% CI. Results for CRP are displayed as geometric means.
